# Treatment of dogs with fluralaner reduced pyrethroid-resistant* Triatoma infestans* abundance, *Trypanosoma cruzi* infection and human-triatomine contact in the Argentine Chaco

**DOI:** 10.1186/s13071-022-05343-2

**Published:** 2022-07-13

**Authors:** Ricardo Esteban Gürtler, Mariano Alberto Laiño, Alejandra Alvedro, Gustavo Fabián Enriquez, Natalia Paula Macchiaverna, María Sol Gaspe, Marta Victoria Cardinal

**Affiliations:** 1 Laboratory of Eco-Epidemiology, Facultad de Ciencias Exactas y Naturales, Universidad de Buenos Aires, Ciudad Universitaria, Buenos Aires, Argentina; 2 Instituto de Ecología, Genética y Evolución de Buenos Aires, Consejo Nacional de Investigaciones Científicas y Técnicas, Ciudad Universitaria, Buenos Aires, Argentina

**Keywords:** Gran Chaco, Vector control, Pyrethroid resistance, Fluralaner, Integrated vector control management, *Trypanosoma cruzi*, Dog, Blood-feeding

## Abstract

**Background:**

Triatomine elimination efforts and the interruption of domestic transmission of *Trypanosoma cruzi* are hampered by pyrethroid resistance. Fluralaner, a long-lasting ectoparasiticide administered to dogs, substantially reduced site infestation and abundance of pyrethroid-resistant *Triatoma infestans* Klug (Heteroptera: Reduviidae) in an ongoing 10-month trial in Castelli (Chaco Province, Argentina). We assessed the effects of fluralaner on vector infection with *T. cruzi* and blood meal sources stratified by ecotope and quantified its medium-term effects on site infestation and triatomine abundance.

**Methods:**

We conducted a placebo-controlled, before-and-after efficacy trial of fluralaner in 28 infested sites over a 22-month period. All dogs received either an oral dose of fluralaner (treated group) or placebo (control group) at 0 month post-treatment [MPT]. Placebo-treated dogs were rescue-treated with fluralaner at 1 MPT, as were all eligible dogs at 7 MPT. Site-level infestation and abundance were periodically assessed by timed manual searches with a dislodging aerosol. Vector infection was mainly determined by kDNA-PCR and blood meal sources were determined by enzyme-linked immunosorbent assay.

**Results:**

In fluralaner-treated households, site infestation dropped from 100% at 0 MPT to 18–19% over the period 6–22 MPT while mean abundance plummeted from 5.5 to 0.6 triatomines per unit effort. In control households, infestation dropped similarly post-treatment. The overall prevalence of *T. cruzi* infection steadily decreased from 13.8% at 0–1 MPT (baseline) to 6.4% and subsequently 2.3% thereafter, while in domiciles, kitchens and storerooms it dropped from 17.4% to 4.7% and subsequently 3.3% thereafter. Most infected triatomines occurred in domiciles and had fed on humans. Infected-bug abundance plummeted after fluralaner treatment and remained marginal or nil thereafter. The human blood index of triatomines collected in domiciles, kitchens and storerooms highly significantly fell from 42.9% at baseline to 5.3–9.1% over the period 6–10 MPT, increasing to 36.8% at 22 MPT. Dog blood meals occurred before fluralaner administration only. The cat blood index increased from 9.9% at baseline to 57.9–72.7% over the period 6–10 MPT and dropped to 5.3% at 22 MPT, whereas chicken blood meals rose from 39.6% to 63.2–88.6%.

**Conclusion:**

Fluralaner severely impacted infestation- and transmission-related indices over nearly 2 years, causing evident effects at 1 MPT, and deserves larger efficacy trials.

**Graphical Abstract:**

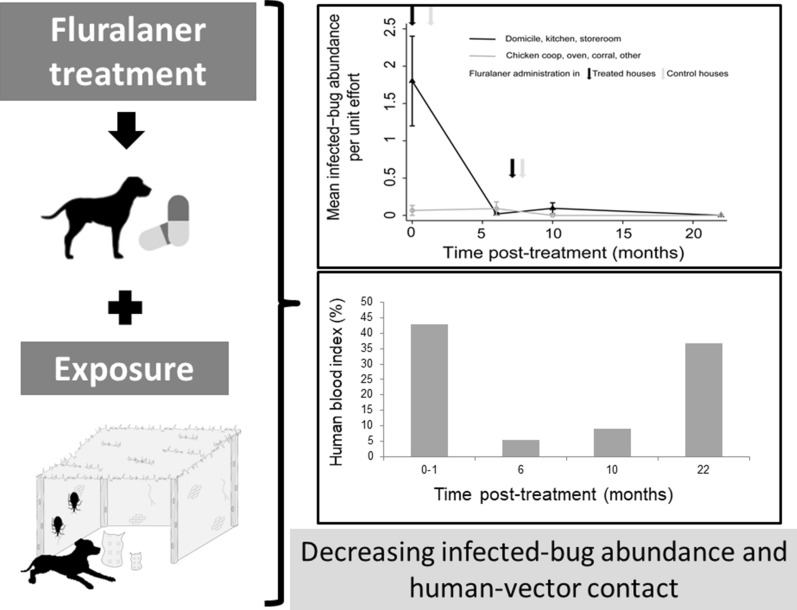

**Supplementary Information:**

The online version contains supplementary material available at 10.1186/s13071-022-05343-2.

## Background

Chagas disease, an important neglected tropical disease caused by *Trypanosoma cruzi*, affects 5–10 million people worldwide, many of whom will become chronically infected, with severe effects on morbidity and life expectancy and increased economic burden [1, 2]. Most human infections are acquired from contact with triatomine bug species that establish persistent colonies in domestic premises or nearby habitats. The advent of synthetic insecticides in the mid-1940s that could be applied with manual compression sprayers were the main tools used in vertical control programs to suppress house infestation with triatomine bugs and prevent human infection with *T. cruzi* [3]. Pyrethroid insecticides have monopolized triatomine control strategies since the mid-1980s, more so since the creation of intergovernmental initiatives during the 1990s that sought to interrupt vector-borne and transfusional transmission of *T. cruzi* at regional scales [4]. Some species were targeted for regional elimination (e.g. *Triatoma infestans*, with the exception of Bolivia, *Rhodnius prolixus* in Central America) and control (e.g. *Triatoma dimidiata* in Central America and *Triatoma brasiliensis* in Brazil) [3–9]. The large-scale implementation of control interventions paved the way to nationwide successes in 11 of 20 endemic countries and within defined sections of other countries [5]. The degree of progress of the regional programs has been widely reviewed [6–9].

One unforeseen obstacle to success in regional elimination efforts was the emergence of pyrethroid resistance in *T. infestans* populations on the Argentina-Bolivia border by the late 1990s and across large sections of Bolivia [10–12]. The immediate solution was to revert to organophosphates (fenitrothion, malathion) and carbamates (bendiocarb) despite their less than optimal safety profiles. Other alternatives investigated, including fipronil, imidacloprip, entomopathogenic fungi and dichlorvos (DDVP)-based fumigant canisters [13], were found to be efficacious against pyrethroid-resistant *T. infestans* but had limited residual activity [3]. Insecticidal paints, including chlorpyrifos, diazinon and pyriproxyfen, provided an effective and longer-lasting option than pyrethroids [14, 15], but organophosphate-based paints have subsequently been restricted or phased out for residential use based on their neurotoxic effects with chronic low-dose exposure. Clearly, new cost-effective tools that can cope with pyrethroid-resistant and peridomestic triatomine populations are needed.

An integrated vector management strategy may benefit from the application of pesticides on nonhuman hosts to kill the bugs that contact or blood-feed on them (i.e. xeno-intoxication) or from drug-based vector control by oral administration of an insecticidal drug. Deltamethrin- or fipronil-impregnated dog collars [16, 17] and spot-on or pour-on formulations of imidacloprid, cypermethrin and fipronil-permethrin administered to chickens, goats, pigeons and dogs were found to kill triatomines up to 1.5 months post-treatment (MPT) [18–21], with more limited residual activity than residual spraying with pyrethroids [3]. Using dogs as both a delivery system and target for control is justified on the basis that they frequently are important blood meal sources [22] and reservoir hosts of *T. cruzi* [23], in addition to being a risk factor for human infection [24].

Fluralaner, an isoxazoline compound that is non-toxic to mammals, effectively kills tick, mites, fleas and sandflies blood-feeding on dogs and poultry (e.g. [25–27]). Modeling studies predicted that fluralaner would reduce mosquito- and sandfly-borne incidence of human disease [28]. In dogs experimentally treated with a single oral dose of fluralaner, early stages of *T. infestans* were killed up to approximately 2 MPT [29]; both susceptible and pyrethroid-resistant late stages of *T. infestans* were killed up to 4 MPT [30]; third or fourth-instar nymphs of *Triatoma brasiliensis* were killed over at least 7 MPT [31]; and adult females of *Rhodnius prolixus* were killed for 3–4 MPT [32]. Our initial results led us to conduct a small placebo-controlled trial of fluralaner administered to outbred healthy dogs in a rural district of the Argentine Chaco with pyrethroid-resistant populations of *T. infestans* [33]. In the fluralaner-treated group, both site infestation and mean abundance dropped significantly to 19–24% of its pre-treatment values by 10 MPT while in the placebo group, both metrics remained stable between 0 and 1 MPT and strongly declined after a rescue treatment with fluralaner to 27–50% of its pre-treatment values [33]. To date, the early and medium-term effects of fluralaner on triatomine abundance combined with *T. cruzi* infection and contact with humans have not been investigated.

House infestation (presence-absence) and the relative abundance of triatomine populations are the traditional metrics used to gauge the immediate effects of control interventions with the goal of reducing or eliminating the target vector species at defined spatial scales. Because the ultimate goal of control programs is to prevent the appearance of new vector-borne infections (i.e. halt parasite transmission) and human disease, the relevant metrics are transmission-related indices based on the occurrence of human infection and other closely related surrogates, such as the relative abundance of *T. cruzi*-infected triatomines in human sleeping quarters (i.e. domiciles or domestic habitats) and infection in companion animals. These more demanding metrics have seldom been used to assess the efficacy of alternate interventions spanning a number of years (e.g. [34–38]). Some disease control programs have measured the degree of progress of large-scale operations across decades using aggregate measures of infestation, total triatomine catch and the prevalence of triatomine infection with *T. cruzi* [39]. Reduction of triatomine abundance may not be enough to interrupt transmission as new human infections with *T. cruzi* may occur when insecticide treatments or other actions fail to fully suppress domestic infestations with *Panstrongylus megistus* [34, 35] or *T. infestans* [36] in rural endemic areas. Therefore, infestation- and transmission-related indices may be required for a full appraisal of intervention effects.

We report here the outcomes of the first field trial of fluralaner administered to dogs with the aim to suppress or reduce transmission indices, including site infestation and triatomine abundance. We assessed the effects of fluralaner on triatomine infection with *T. cruzi*, blood meal sources and abundance of infected bugs stratified by ecotope, and quantified its medium-term effects on site infestation and abundance up to 22 MPT. Following the example set by effective interventions based on community-wide residual spraying with pyrethroid insecticide [40], we hypothesized that fluralaner would strongly reduce the abundance of infected bugs, the prevalence of bug infection and the human blood index (i.e. proportion of enzyme-linked immunosorbent assay [ELISA]-reactive triatomines that had a human blood meal, regardless of whether they contained other identified blood meal sources).

## Methods

### Study area

The field trial was conducted in three small rural villages (Campo Alto, El Asustado and El Ñandú) within the municipality of Juan José Castelli, Chaco Province, Argentina (for description see [41]). Chagas disease vector control personnel repeatedly sprayed rural houses with pyrethroid insecticides during 2000–2014. High levels of pyrethroid resistance was found to occur throughout the municipality [42] and in the houses selected for the present trial [33]. Most houses were constructed with mud walls and metal or thatched roofs, with peridomestic structures (e.g. storerooms, kitchens and chicken coops) housing domestic animals within 5–50 m from human habitations.

### Study design

We conducted a placebo-controlled, before-and-after, nonblinded efficacy trial in 28 sites infested with *T. infestans* divided into two spatially segregated groups as described in [33]. We initially designated a control group based on local triatomines being highly resistant to pyrethroids and there being no other insecticide treatment approved for indoor use. House and site infestations were determined by skilled bug collectors from the Chagas control program and research team members using 15 person-min searches per domestic and peridomestic site with a dislodging spray (0.2% tetramethrin; Espacial, Buenos Aires, Argentina) during March–April 2018, as described in [41]. Householders were informed of the trial goals and effects of fluralaner on ectoparasites, and signed an informed consent form to participate; none refused to do so. Any household that provided informed consent and had at least one infested site anywhere within the premises were eligible to enter the trial. Any dog under 3 months of age or 3 kg of body weight, unhealthy, pregnant and/or aggressive, or which had recently been treated with insecticide was not considered eligible for trial entry.

The treated group included 16 infested sites from the eight infested houses detected in Campo Alto. All dogs in these eight infested households were treated with a single oral dose of fluralaner (Bravecto™; MSD Animal Health, Vicente López, Argentina) according to the manufacturer’s instructions (25–56 mg fluralaner/kg body weight) in mid-April 2018 (set as 0 MPT in both the treatment and control groups). The control group included 12 infested sites from eight infested houses in El Asustado and El Ñandú villages plus one adjacent house from Campo Alto. All listed dogs from these households were treated with a febendazole-based anthelminthic (Antiparasitario Total Nort; Laboratorios Nort, Moreno, Argentina). The 28 infested sites detected prior to the administration of fluralaner were considered to be the target sites for outcome assessment. We photographed all individual dogs and registered their names, age, sex, usual nightly resting site and other relevant data requested from householders.

Dog owners were instructed to tie at least one fluralaner-treated dog to each infested site overnight for at least 2 weeks and to rotate the dogs among sites to minimize stress; they were also requested to remove other domestic animals from the target sites. Householders were provided with a labeled self-sealing plastic bag to collect any triatomine they sighted (i.e. householder collection) for additional evidence of infestation. The distribution of site infestation by ecotope (domicile, storeroom, kitchen, chicken coop and other) and treatment group, and household and demographic features of the dog population have been reported elsewhere [33].

Preliminary results on domestic bug infection supported the administration of a rescue treatment with fluralaner to every dog in the control group at 1 MPT. It should be noted that we retained the denomination of “control houses” for consistency with our prior usage and because the control and treated group differed in pre-treatment bug abundance, leading to two parallel and independent groups [33]. A second treatment round with fluralaner to all eligible dogs (71 of 78) in the 17 study houses was implemented in late November 2018 (7 MPT) for additional control pressure when triatomines resume activity after the cold season. Any eligible dog recruited to the study households was treated with fluralaner. The interventions were well received by householders, and all but one household accepted the offer to re-treat their dogs. The fraction of all listed dogs treated with fluralaner over the previous 4 months (coverage) increased from 46% at 0 MPT to 96, 91, 87 and 0% at 1, 6, 10 and 22 MPT, respectively, with no dog reportedly showing any apparent immediate or delayed hypersensitivity reaction or fluralaner-related adverse effect across the trial [33]. Of the 94 dogs included in the two treatment rounds, 11 (12%) received no fluralaner.

Site-level infestation with triatomines was re-evaluated at each study house at 1, 6, 10 and 22 MPT (i.e. mid-May and mid-October 2018, late February 2019 and February 2020, respectively) using timed manual searches with a dislodging aerosol, as before. Triatomines were also collected after the stipulated search interval (post-timed searches) to increase the number of triatomines for identification of blood meal sources and *T. cruzi* infection. On each survey, we recorded the number of dogs and peridomestic sites in the household and asked householders whether they had sighted any triatomine in their dwellings over the previous period (i.e. householder notification), whether they had applied insecticides (including type of application and insecticide) and/or whether they had modified the structure of target sites.

### Host-feeding patterns

All triatomines collected were identified to species, stage and sex, and preserved as described previously [43, 44]. To determine host-feeding sources, third-instar nymphs and later stages collected by timed searches or householders were dissected, the blood meal extracted in microtubes containing phosphate-buffered saline with crystal violet and tested with a direct ELISA assay against human, dog, cat and chicken antisera with high sensitivity and specificity values [43]. These are the most frequent blood meal sources in the study ecotopes [44, 45]. The tested samples included all triatomines collected in target sites by timed searches in domiciles and up to 20 triatomines per peridomestic site by survey and treatment group, plus 70 triatomines collected by householders and 12 triatomines collected from non-target sites. We report the proportion of reactive triatomines (i.e. those positive by any of the tested antisera) that contained each type of host blood (i.e. host blood index).

### Vector infection

Triatomine infection with *T. cruzi* was determined by kDNA-PCR, except for four insects collected in control houses at 0 MPT, which were microscopically examined at 400× magnification [46]. For molecular identification, we processed up to five third instars or later stages from each target site selected for blood meal identification plus 63 insects collected by householders and 13 triatomines collected from non-target sites. Rectal ampoules were mixed with 50 μl of sterile water and boiled for 10 min, following which parasite DNA was extracted using DNAzol® (Invitrogen, Thermo Fisher Scientific, Waltham, MA, USA) following standard procedures [47]. We used a hot-start PCR targeting a 330-bp amplicon of the kinetoplast minicircle [48]. PCR products were run in a 2% agarose gel and the products visualized under UV using Gel Red® (Biotium, Hayward, CA, USA).

### Data analysis

All data management and statistical analyses were conducted in Stata 15.1 (Stata Corp., College Station, TX, USA). We tested the effects of fluralaner treatment on site-level infestation and triatomine abundance (response variables) relative to 0 MPT using random-intercept logistic (with robust standard errors) and negative binomial regression, respectively, in each cohort of houses taken separately. Odds ratios (OR) and relative abundance ratios (RA) were adjusted for time post-treatment (in months) set as a continuous variable.

Fluralaner effects on host blood indices and vector infection (response variables) over time post-treatment were examined using the Cochran–Mantel–Haenszel (CMH) test. For a meaningful assessment of treatment effects on both variables, we combined the outcomes at 0 and 1 MPT to represent baseline status (to allow for the presumably long induction period of fluralaner via randomly distributed contacts, leading to the eventual survival of pre-treatment insects for 1–2 months and the extended detectability of blood meals by ELISA up to 3 months post-feeding depending on temperature and blood meal size) and pooled subsequent blood meal outcomes from 6 MPT onward as post-treatment status since sample sizes precluded a more detailed analysis. For bug infection, we kept separate observation times over 6, 10 and 22 MPT. These analyses were conducted separately for each main type of ecotope (domiciles, storerooms and kitchens combined vs. other peridomestic habitats mainly or only used by chickens) based on previous studies showing that bug infection was concentrated in the former habitats [46], as were human blood meals. For a summary measure of time-dependent effects of fluralaner treatment on infected-bug abundance across ecotopes, we log-transformed the response variable (after summing 1 unit to every value) and used random-intercept regression as described above.

## Results

### Infestation follow-up

Of the 28 target sites included in the study at 10 MPT, 22 remained in the study until 22 MPT (Table 1, showing the number of target sites in the last two rows). In households with dogs treated with fluralaner (treated group), the overall prevalence of site infestation dropped significantly from 100% at 0 MPT to 19% at 6 MPT and 18% at 22 MPT, whereas mean abundance plummeted from 5.5 to 0.9 to 0.5 triatomines per unit effort over these same time points (Table 1). In control houses, overall site infestation dropped highly significantly from 92%/100% at 0–1 MPT to 50% at 10 MPT and further declined to 18% at 22 MPT; mean abundance fell from 14.7/13.0 triatomines before fluralaner administration to 1.9 at 22 MPT. In domiciles, kitchens and storerooms, the only infested site detected at 22 MPT across groups was a storeroom crowded with chickens, which had a persistent, large infestation across the follow-up period. The other three infested sites at 22 MPT occurred in chicken coops or ovens where chickens nested.

**Table 1:** Site-level infestation and relative abundance of *Triatoma infestans* per unit effort (as determined by timed-manual searches) before and after administration of fluralaner to dogs in the treated group at 0 MPT, dogs in the control group at 1 MPT and dogs in both groups at 7 MPT, by main type of ecotope

Ecotope	Time post-treatment (months)	Control group				Treatment group			
		No. of target sites	Percentage of infested sites^a^	Bug abundance per site		No. of target sites	Percentage of infested sites^c^	Bug abundance per site	
				Mean^b^	SEM			Mean^d^	SEM
Domicile, kitchen, storeroom	0	10	90	16.6	5.9	6	100	4.3	1.3
	1	10	100	14.8	6.5	6	67	2.0	1.1
	6	10	60	4.1	2.4	6	17	1.2	1.2
	10	10	60	4.8	2.8	6	0	0.0	0.0
	22	9	11	2.0	2.0	4	0	0.0	0.0
Chicken coop, oven, corral, other	0	2	100	5.0	3.0	10	100	6.2	2.0
	1	2	100	4.0	3.0	10	30	0.9	0.7
	6	2	50	4.5	4.5	10	20	0.5	0.4
	10	2	0	0.0	0.0	10	30	1.4	0.9
	22	2	50	1.5	1.5	7	29	0.9	0.6
Total	0	12	92	14.7	5.1	16	100	5.5	1.4
	1	12	100	13.0	5.5	16	44	1.3	0.6
	6	12	58	4.2	2.1	16	19	0.8	0.5
	10	12	50	4.0	2.4	16	19	0.9	0.6
	22	11	18	1.9	1.6	11	18	0.5	0.4

Evaluations of house infestation were conducted at 0, 1, 6, 10 and 22 MPT

*CI* Confidence interval, *MPT* months post-treatment,* OR* odds ratio,* SEM* standard error of the mean,* RA* abundance ratio

^a^OR 0.81, 95% CI 0.70–0.92; Wald *χ*^2^ = 9.7, *df* = 1, *P* = 0.002

^b^RA 0.85, 95% CI 0.80–0.90; Wald *χ*^2^ = 27.3, *df* = 1, *P* < 0.001

^c^OR 0.81, 95 CI 0.66–1.00; Wald *χ*^2^ = 3.7, *df* = 1, *P* = 0.05

^d^RA 0.86, 95% CI 0.79–0.94; Wald *χ*^2^ = 11.4, *df* = 1, *P* < 0.001

Householders reported the recent presence of single specimens of *T. infestans* in three of 15 houses by 22 MPT; two houses had been abandoned over the previous year. When asked on recent interventions over the period 10–22 MPT, householders reported partial re-construction of a storeroom containing one target site (negative for *T. infestans* over 10–22 MPT), and residual spraying of pyrethroid with backpack compression sprayers in three houses (with 1 house remaining infested after the reported spraying; one remaining non-infested; and one reverting from infested to non-infested between 10 and 22 MPT). Eighty-one sites were negative for *T. infestans* at 0 MPT and 40 sites appearing later were inspected up to four times over the 22-month study period, involving 454 individual site searches for triatomines; *T. infestans* was detected in only three sites (including a domicile at both 10 and 22 MPT, and a lumber pile used by chickens at 22 MPT).

### Host blood indices

**Fig. 1:**
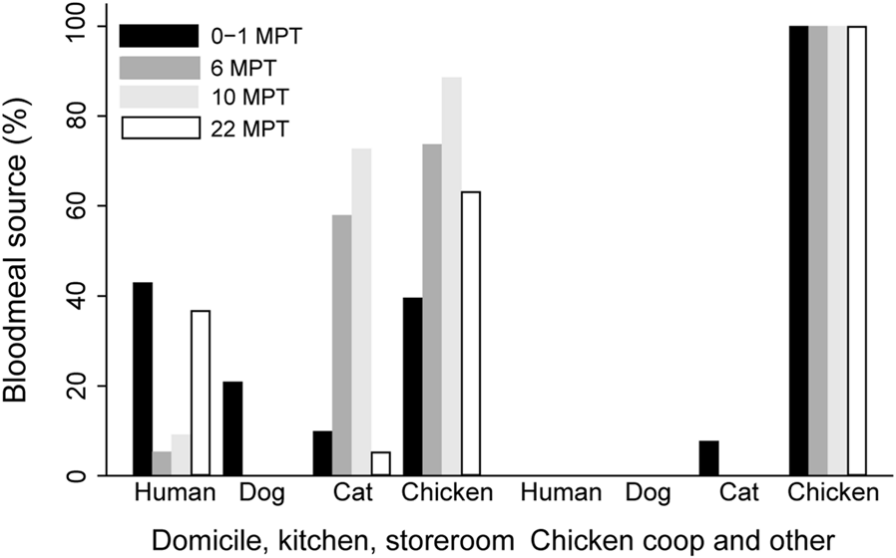
Host blood indices of *Triatoma infestans* (third instars and later stages) stratified by main type of ecotope over time post-treatment of dogs with fluralaner. Abbreviations: MPT, Months post-treatment

In total, 250 (64.9%) of 385 *T. infestans* tested for blood meal sources were found to be ELISA-reactive (Table 2). We analyzed the effects of fluralaner treatment on host blood indices stratified by main type of ecotope (Fig. 1). In domiciles, kitchens and storerooms, the human blood index significantly plummeted from 42.9% at baseline to 5.3% at 6 MPT and to 9.1% at 10 MPT, and then increased to 36.8% at 22 MPT (CMH *χ*^2^ = 21.7, *df* = 1, *P* < 0.001; OR 0.20, 95% CI 0.09–0.42). In domiciles only, human blood meals peaked at 89.5% among 57 reactive triatomines. Dog blood meals only occurred at baseline (20.9%)—before the administration of fluralaner (Fig. 1). The cat blood index steadily and highly significantly increased from 9.9% at baseline to 57.9% at 6 MPT and 72.7% at 10 MPT, and then dropped to 5.3% at 22 MPT (CMH *χ*^2^ = 42.6, *df* = 1, *P* < 0.001; OR 10.89, 95% CI 4.43–26.81). The chicken blood index rose from 39.6% at baseline to 73.7% at 6 MPT and 88.6% at 10 MPT, and slightly declined to 63.2% at 22 MPT (CMH *χ*^2^ = 29.6, *df* = 1, *P* < 0.001; OR 5.49, 95% CI 2.75–10.93). The fraction of triatomines with mixed blood meals significantly increased from 13.2% at baseline to 36.8% at 6 MPT and 70.5% at 10 MPT, and then declined to 5.3% at 22 MPT (CMH *χ*^2^ = 23.7, *df* = 1, *P* < 0.001; OR 5.51, 95% CI 2.54–11.94) (Table 2). In peridomestic habitats occupied by chickens, all reactive bugs (58) had fed on chickens, with two also having fed on cats (Fig. 1).

**Table 2:** Distribution of blood meal sources of *T. infestans* (third instars and later stages) stratified by main type of ecotope over time post-treatment of dogs treated with fluralaner

Ecotope	Time post-treatment (months)	No. of bugs tested	No. of bugs reactive	No. (%) of bugs with mixed blood meals	Host blood source,* n* (%)			
					Human	Dog	Cat	Chicken
Domicile, kitchen, storeroom	0–1	169	91	12 (13.2)	39 (42.9)	19 (20.9)	9 (9.9)	36 (39.6)
	6	53	38	14 (36.8)	2 (5.3)	0 (0.0)	22 (57.9)	28 (73.7)
	10	48	44	31 (70.5)	4 (9.1)	0 (0.0)	32 (72.7)	39 (88.6)
	22	30	19	1 (5.3)	7 (36.8)	0 (0.0)	1 (5.3)	12 (63.2)
Chicken coop, oven, corral, other	0–1	47	26	2 (7.7)	0 (0.0)	0 (0.0)	2 (7.7)	26 (100.0)
	6	4	4	0 (0.0)	0 (0.0)	0 (0.0)	0 (0.0)	4 (100.0)
	10	20	16	0 (0.0)	0 (0.0)	0 (0.0)	0 (0.0)	16 (100.0)
	22	14	12	0 (0.0)	0 (0.0)	0 (0.0)	0 (0.0)	12 (100.0)
Total	0–1	216	117	14 (12.0)	39 (33.3)	19 (16.2)	11 (9.4)	62 (53.0)
	6	57	42	14 (33.3)	2 (4.8)	0 (0.0)	22 (52.4)	32 (76.2)
	10	68	60	31 (51.7)	4 (6.7)	0 (0.0)	32 (53.3)	55 (91.7)
	22	44	31	1 (3.2)	7 (22.6)	0 (0.0)	1 (3.2)	24 (77.4)

### Vector infection

Fifty (10.5%) of 477 triatomines tested positive for *T. cruzi* over the follow-up period (Table 3). The overall prevalence of bug infection steadily decreased from 13.8% at 0–1 MPT to 6.4, 2.9 and 2.3% at 6, 10 and 22 MPT, respectively (Table 3). Most of the infected triatomines detected (41/50, 82%) were collected in domiciles. Vector infection in domiciles, kitchens and storerooms significantly decreased from 17.4% at 0–1 MPT to 4.7, 4.2 and 3.3% at 6, 10 and 22 MPT, respectively (CMH *χ*^2^ = 5.24, *df* = 1, *P* = 0.02; OR 0.58, 95% CI 0.36–0.92). In domiciles only, stage-specific infection rates increased from 0% in second-third instars, 35.3% in fourth instars and 20.0% in fifth instars, to 42.5–56.0% in adult insects. Triatomine infection was rare (3/115, 2.6%) in peridomestic habitats mainly occupied by chickens across the follow-up, and comprised adult stages only.

**Table 3:** Prevalence of *T. cruzi* infection of *Triatoma infestans* (third instars and later stages) stratified by main type of ecotope over time post-treatment of dogs with fluralaner

Ecotope	Time post-treatment (months)	No. of bugs collected^a^	No. of bugs tested	Percentage of bugs tested	Percentage of infected bugs
Domicile, kitchen, storeroom	0–1	371	241	65.0	17.4
	6	64	43	67.2	4.7
	10	53	48	90.6	4.2
	22	69	30	43.5	3.3
Chicken coop, oven, corral, other	0–1	98	77	78.6	2.6
	6	14	4	28.6	25.0
	10	20	20	100.0	0.0
	22	24	14	58.3	0.0
Total	0–1	469	318	67.8	13.8
	6	78	47	60.3	6.4
	10	73	68	93.2	2.9
	22	93	44	47.3	2.3

^a^By timed manual searches, post-timed manual searches and householders

Of 20 ELISA-reactive, *T. cruzi*-infected triatomines collected across the trial, 14 (70%) had fed on humans (including a human-cat blood meal), two had fed on cats only, two had fed on dogs and chicken and two had fed on chickens only. The relative odds of infection among ELISA-reactive triatomines collected in domiciles, kitchens and storerooms was nearly sixfold greater when the insect had fed on humans rather than on any other host (CMH *χ*^2^ = 20.5, *df* = 1, *P* < 0.001; OR 5.57, 95% CI 2.41–12.90).

**Fig. 2:**
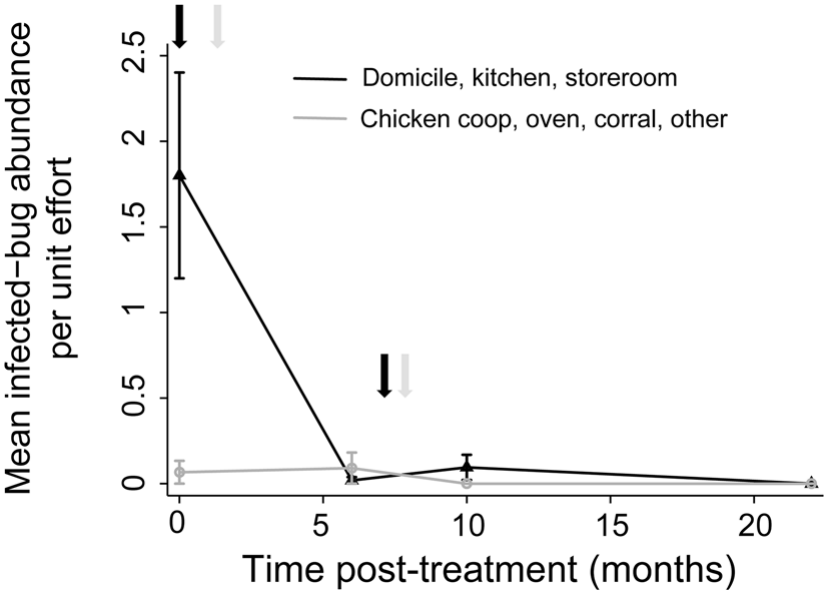
*Trypanosoma cruzi*-infected abundance of *T. infestans* (third instars and later stages) stratified by main type of ecotope over time post-treatment of dogs with fluralaner

Figure 2 shows the distribution of *T. cruzi*-infected abundance of *T. infestans* in the two main types of ecotopes over time post-treatment with fluralaner. In domiciles, storerooms and kitchens, the index plummeted by nearly two orders of magnitude (from 1.80 to 0.02 infected triatomines per unit effort) immediately after fluralaner treatment and thereafter remained marginal (0.10) or nil. In habitats occupied by chickens, infected-bug abundance remained marginal throughout the observation period. Log-transformed infected-bug abundance across ecotopes decreased highly significantly (*P* < 0.001) over time after fluralaner treatment (slope coefficient: − 0.0082, standard error: 0.0023; Wald *χ*^2^ = 12.6, *df* = 1, *P* < 0.001).

## Discussion

Our study shows substantial, prolonged effects of two rounds of fluralaner administered to dogs (at 0–1 and 7 MPT) on site infestation and triatomine abundance, infection with *T. cruzi* and human-vector contact in two cohorts of infested houses over a 22-month period. Mean site infestation and abundance declined at the same rates to converge to very low levels by the trial endpoint in both cohorts despite the heavier baseline infestations recorded in the control group. Other potentially disruptive actions implemented by householders across the trial, especially over the period 10–22 MPT, were minor and most likely ineffectual on site infestation as they were pyrethroid sprays applied to highly resistant triatomine populations and two of them were non-professional applications.

The lack of recovery of *T. infestans* populations after fluralaner effects waned (approximately by 11 MPT) is noteworthy and contrasts with the expected late-summer peak of triatomine abundance [49] and the fast recovery of pyrethroid-susceptible *T. infestans* after community-wide application of pyrethroids in the Argentine Chaco [50]. No rebound was recorded in the fluralaner trial. Consistent with the 4-month duration of fluralaner in the plasma of dogs treated with a single oral dose [51] was the duration of its lethal effects in both pyrethroid-susceptible and pyrethroid-resistant *T. infestans* [30]. The slow population recovery rates recorded in the present study are consistent with experimental evidence showing that compared with pyrethroid-susceptible triatomines, pyrethroid-resistant *T. infestans* have lower fertility and developmental rates [52], delayed excretion or defecation times [53], less tendency for active dispersal [54] and thicker cuticles [55]. These adaptive traits imply fitness costs and may explain the lower triatomine densities than in other rural settings in the dry Chaco infested with pyrethroid-susceptible *T. infestans* (e.g. see [40]), the lack of recovery of bug population size and the very slow propagation to initially negative sites (non-infested before treatment) despite the nearby occurrence of low-density infestations.

Realization of the potential of fluralaner treatment to suppress triatomines is contingent on achieving adequate triatomine–dog contact rates, which are determined by relative host availability and exposure patterns [22]. Nearly all of the dogs included in the study were treated with fluralaner, and roughly 50–65% of them were reportedly tied up to a target site for 3–4 weeks, with other treated dogs ranging freely throughout the compound and nearby areas. The local availability of alternative suitable hosts, especially chickens in some peridomestic habitats, most likely hampered local vector elimination by reducing dog-triatomine contact rates, as recorded previously in experimental and field settings [43–45]. In the present study, the four infested target sites that persisted as such until trial endpoint were occupied by chickens.

Triatomine infection with *T. cruzi* in domiciles, kitchens and storerooms plummeted after the fluralaner treatment of dogs and then remained at 3.3–4.2% over 10–22 MPT, implying lower risks of infection to the residual triatomine populations. Most (82%) of the infected triatomines detected occurred in domiciles, and 70% of these ELISA-reactive triatomines had fed on humans, with bug infection substantially increasing from the fourth or fifth instars to the adult stage. This clearly points to human sleeping quarters as the main habitat where the bulk of transmission occurred and to humans as the main putative source of *T. cruzi*. In a study conducted in a neighboring rural district, human infectiousness to the vector was negatively related to age and aggregated, with 18% of *T. cruzi*-seropositive people generating 80% of triatomine infections [56]. Conversely, bug infection occurred rarely in habitats occupied by chickens and was restricted to adult triatomines that most likely migrated in from domiciles or elsewhere. Although both dogs and cats have frequently been involved in domestic transmission cycles of *T. cruzi* mediated by *T. infestans* and other triatomines of public health significance (reviewed in [22, 23]), the lack of any strong association between individual vector infection and having a dog or cat blood meal suggests that these hosts played a minor role as post-treatment parasite sources in the study houses. A similar inference can be drawn from the substantial increase in cat blood indices post-treatment while triatomine infection decreased to the all-time local minimum.

The abundance of infected bugs, a composite index of site-level triatomine abundance and infection, also plummeted after fluralaner treatment and remained at very low or nil values up to the trial endpoint, implying marginal transmission risks to resident hosts. Multiple studies have reported that mean infected-bug abundance correlated positively and significantly with the household occurrence of human and dog infection with *T. cruzi* in widely diverse settings [24, 35, 46, 57–60]. In our study, the steep decline in infected-bug abundance was directly linked to the suppression of pre-treatment infestations in domiciles (thus killing the infected triatomines) and declining human blood indices rather than to fluralaner having any bug-repellent effect, the lack of which has been demonstrated in independent experimental trials involving three triatomine species [29, 30, 32]. Infected-bug abundance was slightly more closely related to domestic transmission of *T. cruzi* than to abundance-based metrics [58, 59] and should be used more widely to assess the parasitological effects of intervention trials.

Fluralaner administration to dogs disrupted the baseline host-feeding patterns of *T. infestans* and increased the prevalence of mixed blood meals (i.e. host shifts) in domiciles, kitchens and storerooms. At these locations, the human blood index largely fell over the period 0–10 MPT and partially recovered at 22 MPT while both cat and chicken blood indices substantially increased for most of the follow-up period. Elsewhere in this region, the human blood index of domestic triatomines was significantly related to the number of human residents (positively) and dogs or chickens (negatively) [44, 45, 61]. Various fitness-related measures have shown that domiciles and chicken coops are key productive habitats of *T. infestans* populations, with domiciles serving as prime targets of dispersant triatomines and as sources for further propagation [44]. Infestations of chicken coops with *T. infestans* or *Triatoma sordida* and chicken blood meals have traditionally been widespread across rural endemic areas of the Argentine Chaco [45, 61–63]. Cat blood meals, substantially more frequent in the present trial than in other host-feeding surveys in this region [44, 45, 61], first increased post-treatment with fluralaner and then decreased to baseline values. Given the absence of repellent effects of fluralaner on triatomines [29, 30, 32], the large variations we recorded in cat blood indices across the follow-up period accord well with the opportunistic nature of blood-feeding in Triatominae and shifting host availability (e.g. see [22]). While dog blood indices at baseline occurred at roughly similar levels as in other recent studies in this region [44, 45, 61], the subsequent absence of dog blood meals was expected on the basis that: (i) virtually all dogs had been treated with fluralaner and acted as lethal traps over 1–10 MPT; and (ii) very few dog resting sites (domiciles, storerooms and kitchens) were infested after the second treatment round with fluralaner and few triatomines were caught in them.

This study has a number of limitations, of which the most important are the small number of houses, including 28 initially infested sites, non-randomized treatment allocation and lack of a true control group throughout the trial based on ethical reasons. To overcome the absence of a control group we analyzed the data using a before-after design in two spatially segregated, non-randomized clusters of houses. We corroborated the fast effects of fluralaner treatment on triatomine population size and infestation in the treated group only [33]; in the control group (first treated with fluralaner at 1 MPT), the first evaluation of results at 6 MPT precluded estimating the rate at which fluralaner affected the bug population. The use of timed manual searches for outcome assessment also has its limitations. Repeat searches using timed manual searches with a fixed catch effort per site partially compensated for its limited sensitivity [64] and corroborated that non-target sites rarely became infested with *T. infestans* across the follow-up. Removal of triatomines by limited timed searches would not normally affect the viability of established populations of *T. infestans* unless they are very small and the habitat is disassembled to increase the catch. Because established domestic infestations with triatomines tend to persist indefinitely in the absence of effective control interventions (see references in [33]), the probability of ever detecting a truly infested site at least once increases with the number of search occasions when detectability per unit effort remains fixed over time. Moreover, householder bug collections and notifications tended to support the house-level outcome of the timed manual searches in this and other long-term surveillance efforts of house infestation with *T. infestans* [62, 65].

### Conclusions

Fluralaner administered to dogs severely impacted infestation- and transmission-related indices of pyrethroid-resistant populations of *T. infestans* over nearly 2 years, with evident effects recorded as early as 1 MPT. Fluralaner thus appears to be a new tool to use against pyrethroid-resistant and pyrethroid-susceptible triatomines. Whether fluralaner administered to chickens and cats affects triatomines as in dogs remains to be investigated, as does the cost-effectiveness of fluralaner relative to any other potentially effective alternative. If cost-effective, fluralaner treatment of dogs, chickens and cats may be required to fully suppress large infestations in endemic rural settings with a diversity of foci and blood meal hosts, with collateral effects on other ectoparasites and disease vectors. In addition, maximum residue limits of fluralaner are recommended in chickens for human consumption. Xeno-intoxication may be part of an integrated vector management strategy [66] based on community mobilization and supervised implementation of operations. Future research efforts should include cluster-randomized efficacy trials of fluralaner in heavily infested areas [67]; emerging new issues will include setting up appropriate control groups and leaving untreated houses and dogs behind. Larger efficacy trials would also allow larger samples sizes to assess site-level variations in triatomine abundance, blood-feeding patterns and infection with *T. cruzi* in relation to local host availability and infection status.

## Supplementary Information

**Additional file 1: Dataset S1.** Individual- and site-level infestation and triatomine abundance, infection and host-feeding patterns, Castelli, Chaco, 2018–2020.

## Data Availability

Data supporting the conclusions of this article are included within the article. The datasets generated during and/or analyzed during the present study are available in Additional file 1: Dataset S1.

## Abbreviations

CMH: Cochran–Mantel–Haenszel
MPT: Months post-treatment
